# Smart Biosensors for Cancer Diagnosis Based on Graphene Quantum Dots

**DOI:** 10.3390/cancers13133194

**Published:** 2021-06-26

**Authors:** Daniela Iannazzo, Claudia Espro, Consuelo Celesti, Angelo Ferlazzo, Giovanni Neri

**Affiliations:** Department of Engineering, University of Messina, Contrada Di Dio, 98166 Messina, Italy; espro@unime.it (C.E.); ccelesti@unime.it (C.C.); angelo.ferlazzo@unime.it (A.F.); gneri@unime.it (G.N.)

**Keywords:** graphene quantum dots, cancer diagnosis, cancer biomarkers, biosensors

## Abstract

**Simple Summary:**

Graphene quantum dots, the next generation of graphene family, due to their remarkable physical and chemical properties, have been shown to be ideal sensing elements for the early diagnosis of cancer. In this review, we report the recent advances in the synthesis and functionalization of GQDs with different biomolecules able to selectively recognize and convert into a signal specific cancer biomarker, by means of optical, electrochemical and chemiluminescent biosensors. These sensors have shown to ensure the effective and sensitive detection of important cancer biomarkers such as antigens, enzymes, hormones, proteins, cancer related byproducts, biomolecules exposed on the surface of cancer cells and also changes in pH. The developed biosensors can allow the effective diagnosis of cancer diseases as well as the evaluation of the anticancer therapy effectiveness.

**Abstract:**

The timely diagnosis of cancer represents the best chance to increase treatment success and to reduce cancer deaths. Nanomaterials-based biosensors containing graphene quantum dots (GQDs) as a sensing platform show great promise in the early and sensitive detection of cancer biomarkers, due to their unique chemical and physical properties, large surface area and ease of functionalization with different biomolecules able to recognize relevant cancer biomarkers. In this review, we report different advanced strategies for the synthesis and functionalization of GQDs with different agents able to selectively recognize and convert into a signal specific cancer biomarkers such as antigens, enzymes, hormones, proteins, cancer related byproducts, biomolecules exposed on the surface of cancer cells and changes in pH. The developed optical, electrochemical and chemiluminescent biosensors based on GQDs have been shown to ensure the effective diagnosis of several cancer diseases as well as the possibility to evaluate the effectiveness of anticancer therapy. The wide linear range of detection and low detection limits recorded for most of the reported biosensors highlight their great potential in clinics for the diagnosis and management of cancer.

## 1. Introduction

Cancer diseases, characterized by the uncontrolled growth and spread of abnormal cells, are the second leading cause of death globally, behind cardiovascular diseases. The World Health Organization (WHO) estimated 18.1 million new cases in 2018 and 9.6 million cancer deaths [1]. Moreover, based on the projected population growth and aging, the global cancer burden is expected to nearly double in 2040 [2]. The severity of pathophysiological processes related to cancer and the dramatic ever-growing number of cancer deaths has the scientific community to find new breakthrough treatments and new tools for early cancer diagnosis [3,4]. The timely diagnosis of cancer diseases in early stage is a pivotal factor to increase the chances of treatment success and to reduce cancer-related deaths. Nowadays, the preliminary diagnosis is mainly based on high-end methods such as X-ray, nuclear magnetic resonance, ultrasound based imaging, and computed and positron-emission tomography, which may suffer from low sensitivity and/or low resolution and generally low ability to detect small numbers of cancer cells, ideally before the angiogenic switch [5]. The molecular diagnostic tools for the measurements of cancer biomarkers such as the enzyme-linked immunosorbent assay (ELISA), polymerase chain reaction (PCR), mass spectrometry (MS), chromatography and gel electrophoresis, show major advantages over image methods, requiring a thousand times lesser cell numbers [6]. However, these improved detection techniques require time-consuming procedures, skilled personnel, expensive instrumentation and the impracticality to perform the on-site detection in human biological medium, due to the needed sample processing procedures, handling conditions, and storage time, thus often leading to poor outcomes. The recent advances in nanotechnology have demonstrated great promise in revolutionizing cancer diagnostics by empowering miniaturized biosensors for the on-site early and efficient diagnosis [4,7]. Nanobiosensor technologies have a huge potential over conventional methods, such as the real-time analysis, low limits of detection (LOD), high-throughput screening, label-free detection and a small amount of sample to be analyzed [8]. Nanosized materials with dimensions in the range of 1–100 nm, derived from organic or inorganic sources, have been widely investigated to modify electrode surfaces in order to afford biosensors with improved reproducibility, selectivity and sensitivity [9,10]. Their surface modification with suitable functional groups, targeting biomolecules, drugs, and genes, allowed the development of advanced nanoplatforms for various applications in cancer detection and therapy [11]. The used nanomaterials were shown to be biocompatible, structurally suitable for sensors fabrication and endowed of strong adsorption ability [12]. Unlike conventional counterparts used for biosensing, materials in the nanoscale exhibit unique physicochemical characteristics such as quantum size and macroscopic quantum tunnel effect, large surface-to-volume ratios and ease surface functionalization, thus allowing the development of highly sensitive electrochemical, electrochemiluminescent, magnetic, gravimetric and optical biosensors [13]. 

Among the different classes of nanomaterials, the carbon based materials characterized by the presence of graphene structure, such as pristine graphene, graphene oxide (GO), reduced graphene oxide (rGO), carbon nanotubes (CNTs) and graphene quantum dots (GQDs) present several advantages due to their outstanding chemical, physical, and optical properties, leading to ultrasensitive and selective biosensor devices [14,15]. Graphene, the two-dimensional single atomic layer of sp^2^ hybridized carbon atoms, due to its remarkable properties including its large specific surface area, high mechanical strength, thermal conductivity, carrier mobility and electrical conductivity has attracted great interest in the scientific community for the development of low-cost and sensitive electrochemical biosensors and for many biological applications including drug and gene delivery and cancer phototherapy [11,16]. Its oxygenated derivatives, GO and rGO, because of the presence of oxygenated functional groups in their structure, have been conjugated with different types of inorganic nanoparticles, quantum dots, organic polymers and biologically relevant molecules, affording graphene-based biosensors endowed with great sensitivity, selectivity and operational stability, as well as advanced nanocomposites for drug delivery applications [17,18]. CNTs, constituted by graphene sheets rolled into a cylindrical shape, owing to their remarkable electronic and mechanical properties have been also widely exploited for a wide range of in vitro and in vivo applications, ranging from the development of highly sensitive biosensors to smart drug delivery systems for anticancer and antiviral therapy [15,19,20]. From a chemical point of view, the multiple sites of functionalization of these nanomaterials allow the covalent bonding with organic functionalities or biomolecules to the graphene surface or to oxygen groups present on the nanomaterials after oxidative treatments and/or their non-covalent electrostatic interactions, such as the π−π interaction between aromatic compounds and the graphene structure. Moreover, the possibility offered by the multi-functionalization can also allow the concurrent or multiple detection of biomolecules [21].

**Figure 1:**
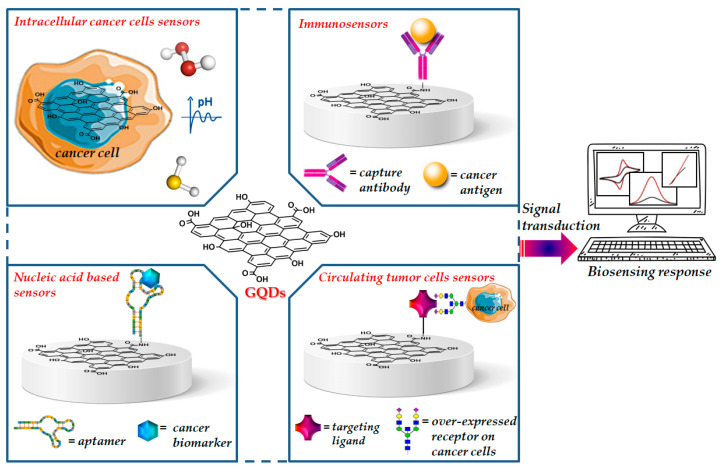
Representative image of functionalized GQDs for cancer biosensing.

Zero-dimensional GQDs, the next generation of the graphene family, received significant interest from academics and industries over the last few years [22]. Their remarkable physicochemical properties enabled novel and extraordinary applications in several fields including materials science, physics, chemistry, biology and medicine [23,24,25]. Unlike two-dimensional graphene, GQDs have a band gap because of quantum size effect and exhibit stable and size-dependent photoluminescence, which can be tuned during their synthesis, by controlling their size shape, doping with heteroatoms and the charge transfers between functional groups and the graphene surface [26,27]. GQDs have been loaded with drugs and tumor-targeting ligand units able to specifically recognize cancer receptors exposed on the cell surface by generating new therapies for efficient targeted delivery of anticancer agents as well as the development of new imaging agents for the in vitro and in vivo diagnosis of several types of cancer [28,29]. Due to their intrinsic optical properties, GQDs have been designed for application in photovoltaics, electro/photo/chemical catalysis, fabrication of flexible devices and biosensing [30]. Recent literature data report the use of GQDs functionalized with functional groups or biomolecules able to recognize cancer biomarkers or to detect tumor cells, as sensing elements for the selective early detection diagnosis of cancer (Figure 1). 

The aim of this review is to report an overview of the recent advances, in the development of different classes of biosensors based on GQDs, for the cancer diagnosis, focusing the attention on the ideal properties of GQDs for biosensing and taking into account the methods used for their synthesis and functionalization procedures, and their possible use in clinics for the diagnosis and management of cancer.

## 2. Suitable Properties of GQDs for Designing Nano-Scaled Biosensors 

**Figure 2:**
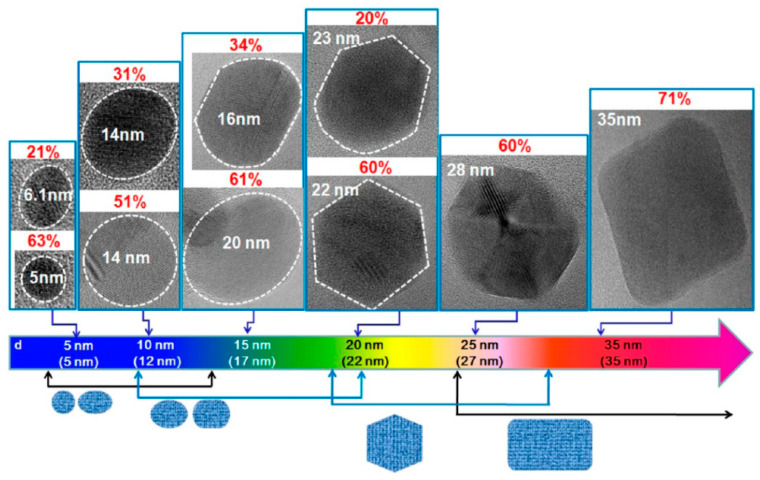
HRTEM images of GQDs reporting their major shapes and the corresponding populations (p) with the increasing average sizes. The dotted lines indicate the region of a GQD, and p is defined as the ratio of number of GQDs with a major shape for each average size. Reprinted with permission from Ref. [35]. Copyright 2012, American Chemical Society.

GQDs, the new class of fluorescent materials from carbon nanomaterials family, possess ideal chemical and physical properties to be used and integrated in sensors for biological and medical applications [31]. From a morphological point of view, GQDs show the peculiar features of both graphene and carbon dots (CDs). CDs and GQDs are zero dimensional carbon based materials, both endowed with unique physicochemical properties associated with quantum size and edge effects. While CDs are mainly synthesized by bottom-up strategies and show spherical shape up to 10 nm, GQDs are typically derived from materials where the sp^2^ carbon atoms are organized into a graphene structure [32]. The presence of a graphene structure and the large surface to volume ratio allow the ease functionalization to a large number of sites of graphene surface. Antibodies, proteins, nucleic acids and polymers have been covalently linked and/or conjugated via π−π interaction to the graphene surface of GQDs, affording high selective and sensitive biosensors able to recognize biomarkers associated with various types of cancers [30,31]. The electronic and optical properties of GQDs make these nanomaterials particularly attractive in optoelectronics. These properties depend on their band gap because of quantum confinement, and are influenced by their size, number of layers, shape and edge configuration [26,33]. As a consequence, these properties can be modulated by using optimal carbon sources and size-controlling synthetic methodologies [34]. Kim et al. investigated the photoluminescence of GQDs, synthesized by various synthetic procedures, from near-ultraviolet to blue region and demonstrated the size-dependent shape/edge-state phenomenon affecting the absorption and PL behaviors of these nanomaterials [35]. As observed by high-resolution transmission electron microscopy (HRTEM), GQDs with sizes below 17 nm showed circular or elliptical shapes, while GQDs larger than 17 nm exhibited polygonal shape with the armchair edges (Figure 2). The authors observed the variation in absorption peak, which is consistent with the quantum confinement, whereas the PL peak energies exhibited non-monotonic behaviors as the size increased to 17 nm. This anomalous PL behavior was attributed to the size-dependent shape and to the corresponding edge variations of GQDs, along with the scattering mechanism of graphene.

Most of the reported PL spectra of GQDs show colors ranging from blue to yellow [36]. This limiting narrow spectral coverage can be expanded by doping the energy donors GQDs with heteroatoms or functionalizing their surface [37]. The heteroatom doping of GQDs have been shown to expand the spectral coverage of GQDs to all visible wavelengths, improving their quantum yield (QY) and prolonging their fluorescence lifetime [38]. The ex situ surface functionalization has also proved to enrich their intrinsic state emission, thus opening new possibilities in the use of these zero-dimensional nanomaterials for various biomedical applications including biosensing and bioimaging [39].

Taking advantage of the electrochemical properties similar to graphene, GQDs have been widely used as active electrode materials in the field of electrochemical sensors [40]. The electrochemical platform is simple and smart, allowing it to easily perform a series of electrochemical techniques, such as cyclic voltammetry (CV) and differential pulse voltammetry (DPV) and amperometric (AMP) measurements. The high performance of the GQDs-based electrochemical biosensors is related to the large surface-to-volume ratio, the abundance of hydrophilic/hydrophobic edges/basal planes, as well as the high conductivity of GQDs. The smart combination of chemiluminescence and electrochemistry led to a valuable analytical detection method known as electrochemiluminescence (ECL). ECL active GQDs are expected to have promising applications in the development of novel ECL biosensors, providing high sensitivity, good precision and acceptable stability [41]. Based on these considerations, in the following paragraphs, the relevant role of the employed synthetic methodologies to drive GQDs towards improved biosensing devices will be discussed. 

## 3. Recent Approaches for the Synthesis of GQDs as Biosensing Elements

In order to obtain GQDs with ideal chemical, optical, and electronic properties for biosensing applications, several synthetic strategies have been reported [42,43]. In general, the methods for the synthesis of GQDs are classified into two categories, namely, top-down and bottom-up routes [23]. Some typical strategies are listed in Table 1. Top-down methodologies are based on the cutting and exfoliation of bulk graphene based materials such as graphite, graphene, GO, CNTs, carbon nanohorns, and carbon fibers by chemical or physical treatments, which include chemical exfoliation, hydrothermal and electrochemical methods [42,44,45,46]. These strategies are advantageous for the easily available starting materials and simple operation procedures, and generally afford to GQDs possessing many oxygen-containing functional groups, useful for subsequent functionalizations with bioactive molecules; however, these methods mainly suffer from low yields and lack of morphological control. 

The bottom-up strategies start from small aromatic compounds or other natural or synthetic carbon sources through several methodologies, which include stepwise solution chemistry, hydrothermal and solvothermal procedures, pyrolysis, microwave assisted methods, UV and electron beam irradiation [42,44]. These latter methods allow a more accurate control on the morphology, size, shape and generally afford to nanomaterials endowed with superior optical properties; conversely, these strategies are hindered by the need of expensive aromatic precursors and by complicated stepwise pathways.

### 3.1. Top-Down Approaches 

The top-down methodologies for the synthesis of GQDs can be described as “defect-mediated fragmentation processes”. In these synthetic strategies, the formation of defects in the materials surface induce the tunable evolution of oxygen functional groups, which in turn can act as reactive sites, allowing the formation of very small graphene fragments [81]. The size, shape and amount of oxygen containing groups depend on the used carbon precursor and cutting methodology. The hydrothermal techniques for the synthesis of GQDs starting from graphene based materials are generally based on the use of strong oxidizing agents such as H_2_SO_4_, HNO_3_ and H_2_O_2_, affording nanodots with a high degree of oxygen containing groups [82]. GQDs synthesized following these procedures have been employed for different biosensing applications such as the detection of biomarkers of depression [47], the selective sensing of mercury ions [48] and H_2_O_2_ [49]. Hydrogen peroxide as etching agent and ammonia were also used for the synthesis of GQDs using GO as the starting material. The obtained GQDs have been used as basic components of a surface-enhanced Raman scattering (SERS) substrate for the detection of banned dye pollutants [50]. GQDs have also been successfully synthesized by employing biomass carbon sources such as corn powder [51], cotton cellulose [52] or rice husk biomass [53], and were employed as down conversion materials in dye-sensitized solar cells (DSSCs), in bioimaging and for Fe^3+^ sensing, respectively. 

One of the most investigated methods for the synthesis of graphene based materials is the chemical exfoliation of layered bulk materials by chemical reactions involving intercalants such as acids, bases, inorganic salts, oxidizing agents, and reactive functional molecules [83,84,85,86,87]. Liu et al. synthesized GQDs with pure sp^2^ carbon crystalline structure by chemical exfoliation of graphite nanoparticles in organic solvents [54]. A one- and two-step chemical exfoliation route using KMnO_4_ was also investigated for the synthesis of GQDs starting from graphite flake powders and carbon nanotubes affording nanomaterials with different oxidation levels and PL excitation performances [55]. An ultrasound assisted liquid exfoliation method was used by Sarkar et al. for the synthesis of photoluminescent GQDs starting from graphite powder and using ethyl acetoacetate as solvent, in a strong basic medium [56]. Zdrazil et al. synthesized in high yields, low defect blue fluorescent GQDs by micro-wave expansion and liquid phase exfoliation of graphite [57]. Electrochemical methods, through the use of selective oxidation and reduction reactions, allow to obtain GQDs with accurate size control, in simple, efficient and green reaction conditions [58,59,61,88]. A simple approach to the synthesis of GQDs with blue to green photoluminescence by electrochemical exfoliation of graphite rods was reported by Bahadur et al. [58]. An environmentally friendly, electrochemical synthesis of glycine functionalized GQDs based on the direct exfoliation and oxidation from graphite rods was reported by Zhi et al. [61]. The synthesized bright yellow nanomaterials showed the ability to detect Fe^3+^ ions in a selective manner. In a recent work, Kalita et al. reported the electrochemical synthesis of GQDs with uniform size for the fabrication of soil moisture sensors [59]. Uniform-sized phosphorus and sulfur co-doped GQDs (P, SGQDs), were synthesized by the one-step electrolysis of graphite rods [61]. The synthesized GQDs were used as (ECL) immunosensors for the detection of okadaic acid in mussel samples. An environmentally friendly and single-step electrochemical synthesis of GQDs using as carbon source wood charcoal was reported by Kumar et al. [62]. The synthesized nanomaterials GQDs were investigated for the development of a colorimetric biosensor for the detection of H_2_O_2_ and glucose simultaneously. A green and scalable microplasma assisted electrochemical method for the synthesis of excitation-independent GQDs was recently reported by Joffrion et al. [60]. In this process, GQDs were exfoliated from a graphite rod, used as electrode in the electrochemical cell containing a glucose–water solution and assisted via microplasmas, thus affording GQDs with emission wavelength between violet and red, useful for photonic and electronic applications. 

### 3.2. Bottom-Up Approaches

GQDs with controllable sizes, morphology and optical properties have been produced for biosensing applications by advanced bottom-up methods starting from small aromatic precursors or other carbon sources even originated by the degradation of bulk materials [41,42,43,44,45,46,47,48,49,50,51,52,53,54,55,56,57,58,59,60,61,62,81,82,83,84,85,86,87,88,89]. The most used bottom-up approaches for the synthesis of GQDs as sensing platforms can be classified in hydrothermal methods also assisted with microwave, template methods, and pyrolytic processes. 

A gram-scale synthesis of fluorescent and water soluble GQDs based on a water-phase molecular fusion route under mild hydrothermal conditions was reported by Wang et al. [63]. The synthesized nanomaterials showed highly efficient excitonic fluorescence, strong excitonic absorption bands, large molar extinction coefficients and long-term photostability. In a similar way, Fan et al. reported a large-scale method to synthesize fluorescent and water-soluble GQDs with targeted imaging property by water-phase molecular fusion using pyrene and polyethyleneimine as precursors [64]. In order to improve the optical properties of GQDs, Xi et al. developed the synthesis of boron doped GQDs by a hydrothermal one-step molecular fusion between 1,3,6-trinitropyrene and borax in sodium hydroxide [65]. The synthesized B-GQDs were investigated as fluorescence probes for the detection of Fe^3+^ ion, phosphate and cytochrome C. Choi et al. synthesized cysteine–functionalized GQDs through an hydrothermal process involving the carbonization of citric acid [66]. The produced green fluorescent GQDs were used for the selective Hg^2+^ ion detection. High-yield green-photoluminescent GQDs have been also produced by a hydrothermal method using only glucose and deionized water as precursors [68]. Dai et al. reported a green and one-pot hydrothermal method for the synthesis of GQDs using as reactants the natural polymer starch and water [70]. The synthesized nanodots have been successfully applied to cell imaging. Microwave assisted hydrothermal methods have been also used to combine the benefits of the hydrothermal technique with the advantages offered by microwave irradiation [67,69,71,90]. A microwave assisted hydrothermal method using glucose as precursor and without involving any harmful chemical in the process was recently investigated by Ray et al. for the synthesis of GQD-Au hybrid nanoparticles [71]. The synthesized GQDs were used for the reduction of HAuCl_4_, affording luminescent GQD-Au hybrid nanoparticles. Fresco-Cala et al. reported a one-pot microwave-assisted hydrothermal reaction starting from urea and glucose, in the presence of phosphoric acid [91]. 

Soft template methods are low cost and environmentally friendly procedures. These methods do not require separation and purification procedures, are suitable for large-scale synthesis, and afford nanodots with controllable and uniform size [72,73,74,91]. Yang et al. synthesized GQDs by a soft-template approach using as carbon source and template 1,3,5-triamino-2,4,6-trinitrobenzene (TTAB) [73]. The produced nanomaterials exhibited green fluorescence, good solubility in water, and excellent biocompatibility, thus promoting their use in bio-imaging and optoelectronics. Monodispersed GQDs with strong blue PL emission have been synthesized through the soft-template method using as precursor hexa-*peri*-hexabenzocoronene (HBC) [74]. Gao et al. used carbon disulfide as precursor to synthesize fluorescent GQDs doped with sulfur [75]. 

The direct pyrolysis of small molecules has proved to be a facile bottom–up procedure to synthesize GQDs with homogenous size distribution [75,76,77,78]. Zhao et al. developed a simple synthetic method for the synthesis of GQDs by carbonizing, L-glutamic acid (Glu), using a heating mantle device [77]. The synthesized nanodots, proved to catalyze the reduction of H_2_O_2_ in the presence of a peroxidase substrate. Naik et al. reported a single-step synthesis of GQDs using the pyrolysis of citric acid, at different pH [78]. The authors found that the intensity of the fluorescence decreased with the increase of pH. Blue-photoluminescent GQDs for cell imaging have been synthesized by Chen et al. through pyrolysis of trisodium citrate [79]. 

The irradiation methods used for the synthesis of GQDs represent green, simple and fast approaches for the large scale synthesis of these nanomaterials [79,80,92]. Zhu et al. reported a green approach through the free-radical polymerization reaction of the oxygen-containing aromatic compounds, salicylic acid and pyridine-2, 6-dicarboxylic acid, through ultraviolet irradiation [80]. In a recent work, Devendrappa et al. synthesized reduced GQDs (rGQDs) by using 365 nm UV irradiation for different hours to reduce oxygen containing glucose through a microwave assisted hydrothermal method [92]. The electrochemical behavior of the synthesized nanomaterials demonstrated the potential of rGQDs as active electrode materials. Wang et al. reported a room-temperature method for the synthesis of fluorescent and single-crystalline GQDs by electron-beam irradiation of 1, 3, and 6-trinitropyrene in aqueous solutions of hydrazine hydrate, obtaining GQDs suitable for optoelectronics applications [93].

## 4. Biosensors Based on GQDs for Cancer Detection

Biosensors for cancer diagnosis are designed to recognize and convert into signal, specific cancer analytes such as antibodies, proteins, carcinoma antigens, nucleic acids, cancer related byproducts such as H_2_O_2_, proteins exposed on the surface of cancer cells and changes in pH [4,9,93]. Recently, different strategies involving GQDs as sensing nanoplatforms have been developed for the early, fast and sensitive diagnosis of cancer (Table 2). The reported diagnostic tools have been shown to ensure the effective detection and monitoring of cancer diseases as well as the possibility to evaluate the effectiveness of anticancer therapy, thus demonstrating their great potential in clinics for the diagnosis and management of cancer. In the following paragraphs, we have summarized the most advanced strategies for the development of cancer biosensors based on the use of GQDs, by classifying the reported biosensors as intracellular cancer cell sensors, immunosensors, nucleic acid hybridization sensors and circulating tumor cell sensors.

### 4.1. Intracellular Cancer Cells Sensors

A universal strategy for detecting cancers of different origins could be achieved by using pH probes able to discriminate the differences in cellular environments of healthy or cancer cells. This difference in pH is due to the Warburg effect, leading to the lactic acid accumulation in rapidly growing tumor cells. In fact, because of acidosis, the pH values of healthy cells and tissues, usually found between 7.0 and 7.4, in solid tumors, regardless of their origin, decreases to pH values in the range of 6.4–6.8 [118]. Several authors demonstrated the change of photoluminescence observed in GQDs in response to change of pH in cellular medium [94,95]. Kumawat et al. reported the use of photoluminescent GQDs synthesized from grape seeds, for the effective intracellular pH mapping, in order to understand the various physiological phenomena occurring in the cancer cell microenvironment [95]. The authors demonstrated the ability of nanomaterials to enter cell nucleus in different cell lines (L929, HT-1080, MIA, PaCa-2, HeLa, and MG-63 cells), thus acting as a potential nucleus labelling agent. Optical sensing measurements, showed a gradual decreasing pattern in the PL intensity over pH 2 to pH 13 with response time of ~1 min and sensitivity of −49.96 ± 3.5 mV/pH, thereby demonstrating the potential of this system as a pH sensing agent. A pH-responsive fluorescent system based on sulfur-nitrogen-doped GQDs able to exhibit a sharp fluorescence transition between green (at pH below 6.8) and blue (at pH above 6.8) was reported by Fan et al. [119].

The GQDs used in this study were produced by an electrochemical method, starting from graphite rods in sodium p-toluenesulfonate acetonitrile solution, leading to nanoparticles with uniform size (average diameter of 4 nm) and high photostability. The authors speculated that in weak acidic environment, the protonated nitrogen present on the GQDs surface act as H-bond donor to form an intramolecular N-H-O hydrogen bond, which then interact with the oxygens of the adjacent sulfonic group; this hydrogen bond interaction led to the observed fluorescence switch. The ability of the nanomaterials to detect cancers of different origin at an early development stage was demonstrated by fluorescence imaging in mice bearing PANC-1, HepG2, A549, U87MG, and HeLa tumors. The reported PL emission from the tumor site, due to EPR effect, and the observed upconversion photoluminescence further highlighted the great potential of the system as a universal probe for image-guided surgery and cancer diagnosis. GQDs-based biosensors designed for cancer diseases include biosensors able to detect abnormal levels of small molecules, such as H_2_S or NO, which are normally related to several diseases, such as cancer, diabetes, liver cirrhosis and Alzheimer’s disease [119,120]. Li et al. reported a fluorescent probe based on GQDs synthesized from pyrene, and covalently conjugated with 2,4-dinitrophenoxy-tyrosine (DNPTYR) for the real-time imaging of the intracellular level of H_2_S triggered in response to stimulus [96]. This sensor worked like a turn-on probe for H_2_S, since the presence of this analyte was able to cleave the dinitrophenoxyl group, thus restoring the photoluminescence of the nanomaterial. The authors demonstrated in vitro the ability of this biocompatible and photostable system to measure dynamically in breast cancer cell line (MCF-7), the amount of H_2_S at a concentration as low as 2 nM. The Fe^3+^ ion is involved in different physiological and pathological processes, including anemia, liver damage, diabetes, neurodegenerative disorders and cancer [121]. In order to detect the concentration of Fe^3+^ in living cancer cells, Voelcker et al. synthesized a turn-on orange-red fluorescent biosensor based on rhodamine B functionalized GQDs (RBD-GQDs). The authors found for this fluorescent sensor, detection limits in pancreatic cancer stem cells (CSCs) as low as 0.02 μM [97]. Moreover, after binding with the ion, the RBD-GQDs nanosystem showed orange-red fluorescence with 43% quantum yield, thus suggesting its possible use a labelling agent for cancer cells. 

### 4.2. Immunosensors

Immunosensors are analytical devices able to measure signals in response to specific antibody–antigen interactions. This class of biosensors represent a valuable alternative to the clinically used tests for the detection of biomarkers such as the enzyme-linked immunosorbent assay (ELISA), which requires several separation steps and time-consuming processes. A large number of ultrasensitive and selective immunosensors have been developed using different kinds of transducers, exploiting changes in mass, heat, electrochemical or optical properties [122]. GQDs represent promising nanomaterials for biosensors development due to their large surface area, excellent electrical and thermal conductivity, possibility of doping with heteroatoms and the presence of several oxygen-containing functional groups that allow the formation of stable chemical bonds with different biologically relevant molecules [30,40]. Wu et al. developed an immunosensor for the detection of the ovarian cancer biomarker CA-125, by exploiting the chemiluminescence resonance energy transfer (CRET) to GQDs [98]. In this study, the nanodots synthesized by photo-Fenton method from GO were immobilized on amino-modified glass chips and covalently linked to the capture antibody (cAb), specific for the CA-125 antigen by amide conjugation. The signal transduction from the water soluble chemiluminescent reagent to the GQDs was based on CRET mechanism. The mechanism involved the use of the enzyme horseradish peroxidase (HRP), which in the absence of CA-125 antigen catalyzed the production of the reactive oxygen species (ROS) from H_2_O_2_ and oxidized luminol, thus generating the excited electrons. Then, when the electrons returned to the ground state, chemiluminescence occurred, emitting blue light. When the CA-125 antigen was present in the immunoassay, the formed antibody–antigen complex was exposed to Ab–HRP to form a sandwich structure composed of GQDs–cAb/CA-125/Ab–HRP. In this way, HRP is in close proximity to the GQDs, enabling the resonance energy transfer from the dianion of luminol to the GQDs and quenching the chemiluminescence. The biosensor showed a wide linear range of detection from 0.1 U mL^−1^ to 600 U mL^−1^ with a LOD of 0.05 U mL^−1^ for CA-125. In a recent work, Hasanzadeh et al. investigated the same antigen for the early detection of ovarian cancer in human plasma samples through the development of an electrochemical immunosensor [99]. In this study, nanodots synthesized through thermal pyrolysis of a mixture of citric acid and D-penicillamine (DPA-GQDs) were functionalized with silver nanoparticles and deposited on a GCE while the antibody of CA-125 was covalently linked to the conductive nano-ink modified with cysteamine functionalized Au nanoparticles (CysA) through a covalent bond between the carboxyl group of the antibody and the amine group of CysA–Au. The synthesized nano-ink Ag–DPA–GQDs was used to investigate the formation of an antigen–antibody complex, which was monitored using the differential pulse voltammetry (DPV) technique. The authors obtained a linear range of 0.001–400 U mL^−1^ and LOD of 0.001 U mL^−1^. 

**Figure 3:**
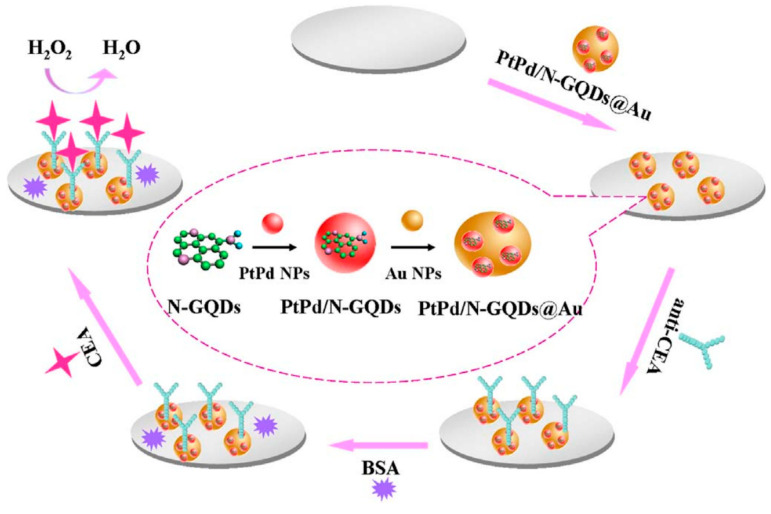
Label-free electrochemical immunosensor PtPd/N-GQDs@Au. Reprinted from Biosensors and Bioelectronics, ref. [100], copyright (2017), with permission from Elsevier.

The carcinoembryonic antigen (CEA) is an oncofetal glycoprotein with a molecular weight of 200 kDa, generally expressed by mucosal cells and over-expressed in various cancer diseases. This antigen represents one of the major tumor biomarkers used for the diagnosis of tumors, such as breast, lung, pancreatic, liver, colorectal, and gastric cancers [123]. The use of electrochemical sensor for CEA detection represents the most effective method to detect this antigen because of the high sensitivity, rapid response and easy to use. Yang et al. reported a ultrasensitive label-free electrochemical immunosensor for CEA detection, using nitrogen-doped GQDs supported PtPd nanoparticles and gold nanoparticles (PtPd/N-GQDs@Au) [100]. In this study, N-doped GQDs, synthesized from citric acid and dicyandiamide by hydrothermal method, were used for the stepwise self-assembly with the bimetallic nanoparticles and then, after amine functionalization, with the gold nanoparticles. The nanocomposite was deposited on a GCE and conjugated with anti-CEA by chemical bonding between PtPd NPs and the amine groups of antibody while BSA solution was used to suppress nonspecific binding sites between the antigen and the surface of electrode (Figure 3). 

The electronic interactions between NGQDs and the metallic nanoparticles contribute to the excellent electrocatalytic activity towards H_2_O_2_ reduction. The nanosystems were applied as transducing materials to efficiently conjugate capture antibodies and to amplify electrochemical signal by analyzing different concentrations of CEA in serum samples. The authors reported a wide linear range (from 5 fg/mL to 50 ng/mL) and a very low detection limit (2 fg/mL). CEA antigen was also investigated as a cancer biomarker by Nie et al. by developing a sandwich-type electrochemiluminescence immunosensor [101]. The authors reported a signal amplification strategy based on poly(5-formylindole)/ reduced GO nanocomposite and Au nanoparticle decorated GQDs (GQDs@AuNP) as electrochemiluminescent probes. The GO nanocomposite facilitated the ion transport during the redox reactions and was used to immobilize the primary antibody (Ab1), while the Au decorated GQDs were loaded with the secondary antibody, which improved the electron transfer capability with stable intensity. Owing to the multiple signal amplification properties, this sandwich-type immunosensor showed good stability, reproducibility, specificity, a wide linear range of sensitivity for CEA detection (from 0.1 pg/mL to 10 ng/mL) and a LOD of 3.78 fg/mL in human serum. Recently, Ganganboina et al. reported a label-free impedimetric immunosensor based on nitrogen and thiol-doped GQDs (N,S-GQDs) with size of 2–9 nm, synthesized by hydrothermal method from thiourea and citric acid [102]. The nanomaterials were used to decorate gold-embedded polyaniline (Au–PANI) nanowires by Au-thiol interaction and were covalently bound with anti-CEA antibody onto the Pt electrode. The detection principle was based on the change in impedance of the nanosystem after interaction with CEA; the formation of the CEA antibody-antigen complex showed to significantly increase the charge transfer resistance, thus affording a label-free immunoassay substrate for the impedimetric CEA detection. To evaluate the feasibility of the nanosystems for real-time biomedical application, the authors determined the antigen in commercial human serum samples. This immunosensor showed a wide linear range of detection (from 0.5 to 1000 ng/mL) with a LOD of 0.01 ng/mL. 

**Figure 4:**
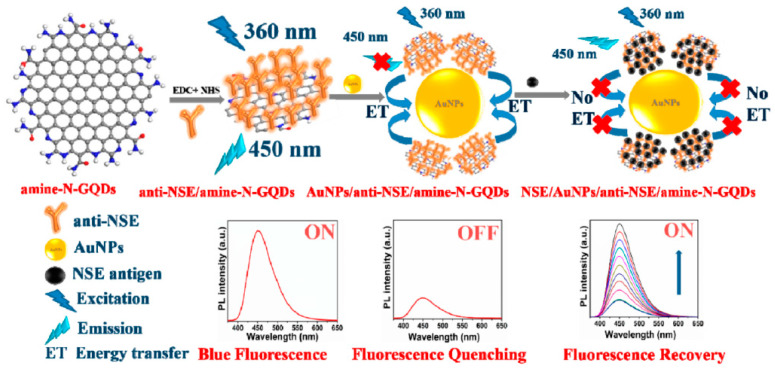
Mechanism of the anti-NSE/amine-N-GQDs@AuNP biosensor for small cell lung cancer biomarker detection. Reprinted with permission from ACS Applied Bio Materials, ref. [39]. Copyright (2020), American Chemical Society.

The neuronspecific enolase (NSE) is a specific serum biomarker for the early diagnosis of small cell lung cancer (SCLC) [124]. Recently, Packirisamy et al. developed a fluorescent turn-on biosensor for the ultrasensitive detection of NSE based on functionalized GQDs as the energy donor system and gold nanoparticles (AuNPs) as the energy acceptor (anti-NSE/amine-N-GQDs@AuNP) [39]. The amine-functionalized and nitrogen-doped GQDs with an average size of ~3 nm were synthesized by hydrothermal method starting from citric acid and diethylenetriamine (DETA) and covalently conjugated to the monoclonal NSE antibody (anti-NSE). The mechanism of this fluorescent biosensor is based on the nanosurface energy transfer (NSET) between these antibody conjugated GQDs and the AuNPs mixed with the nanosystem (Figure 4). The blue fluorescence emission of the anti-NSE/GQDs system (ON state) was quenched by AuNPs, acting as highly efficient fluorescence acceptors (OFF state). The subsequent additions of NSE antigen to the composite solution increases the distance between the antibody conjugated system and the AuNPs, thus allowing the restoration of fluorescence (ON state). The composite showed a broad linear detection range (0.1 pg mL^−1^ to 1000 ng mL^−1^), and a low LOD (0.09 pg mL^−1^). Moreover, when this fluorescent biosensor was investigated in human serum samples, an excellent performance with an average recovery value of 94.69% was reported.

**Figure 5:**
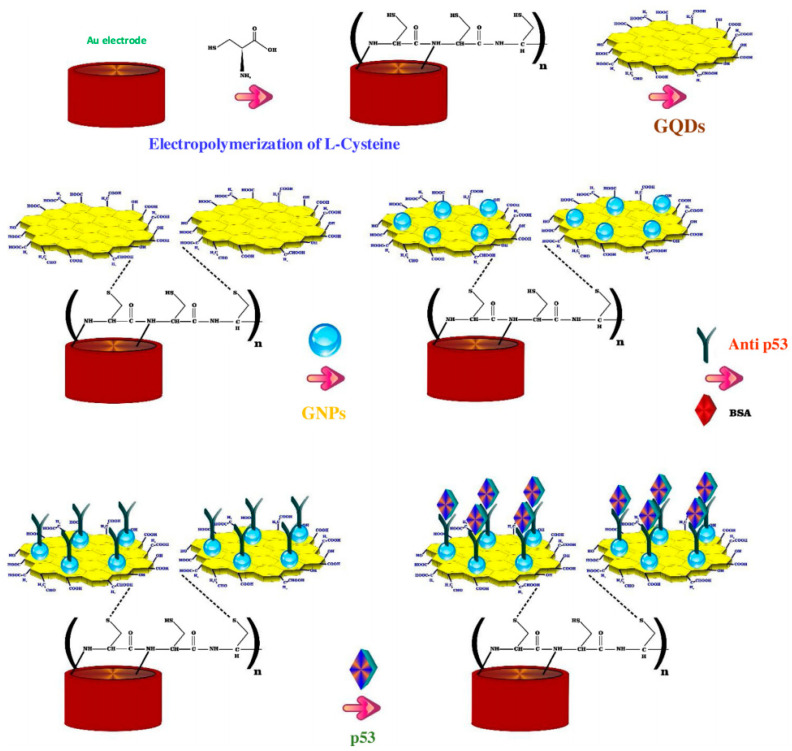
Fabrication steps of the label free electrochemical immunosensors for tumor suppressor protein p53 detection. Reprinted from International Journal of Biological Macromolecules, ref. [104], copyright (2018), with permission from Elsevier.

CA15-3 is the most commonly used tumor marker for the diagnosis of breast cancer [125]. Hasanzadeh et al. developed a label-free immunosensor based on gold nanosphere (Au NSs) electrochemically assembled on thiolated GQDs (CysA/Au NSs/GQDs) for the detection of CA 15-3 [103]. In this work, GQDs synthesized by pyrolyzing citric acid, were electrochemically deposited modified onto GCE and then functionalized with thiol groups and covalently loaded with gold nanoparticles by electrochemical assembly. The so developed nanosystem with 80–140 nm diameter and 20 nm height, provided a large surface area for the effective loading of CA 15-3, thereby increasing the number of binding events occurring between the antigen and antibodies. The authors found that under optimized experimental conditions, the immunosensor demonstrated good sensitivity and specificity, showing, in human plasma sample, a linear dynamic range of 0.16–125 U/mL and a LOD of 0.11 U/mL. Moreover, the application of the biosensor performance to the assay of CA 15-3 malignant cell line lysates of human breast adenocarcinoma (MCF-7 cell line) showed peak currents linearly proportional to the MCF-7 cells concentration, thus highlighting this system as a valuable tool for the point-of-care diagnosis of breast cancer. The same research group, developed a ultrasensitive electrochemical immunosensor based on ternary signal amplification strategy, for the quantitation of the tumor suppressor protein p53 [104]. This protein is involved in many cancer types and is known to be a negative regulator of cell growth [126]. It was demonstrated that its alteration or inactivation by mutation or by interactions with oncogene products of DNA tumor viruses, can lead to cancer [127]. In this study, GQDs with a size distribution of ~5 nm, synthesized by pyrolisis of citric acid, were electrically deposited on the surface of poly-cysteine modified Au electrodes via interaction of the NH_3_^+^ groups of cysteine with the oxygen-containing groups of GQDs. The subsequent sono-electrodeposition with GQDs/AuNPs (GNPs) allowed the covalent immobilization of the biotinylated p53-antibody to the nanocomposite film constituted by poly L-cysteine (P-Cys) as conductive matrix and GNPs as synergetic amplification element (P-Cys-GQDs-GNPs) (Figure 5). The biosensor was then used for the detection of p53 protein by using differential pulse voltammetry (DPV) and square wave voltammetry (SWV) techniques. The authors reported a linear range of detection (0.000592–1.296 pM) and a low LOD of p53 in unprocessed human plasma (0.065 fM). The biosensor performance was also evaluated towards both normal and malignant cell line lysates (the normal cell line from mouse L929, the colon cancer cells HCT, the prostate cancer cell line PC-3, and the human breast adenocarcinoma cell line MCF7), showing excellent sensitivity and long-term stability.

Over-expression of interleukin-13 receptor α2 (IL13Rα2) is observed in several cancer diseases such as glioma, colorectal and pancreatic cancer [128]. Recently, Serafín et al. reported an integrated amperometric electrochemical immunosensor for the determination of IL13Rα2, using an hybrid material composed of multiwalled CNTs (MWCNTs) and GQDs, synthesized by direct pyrolysis of citric acid [105]. The biotinylated IL13Rα2 antibody was covalently linked to streptavidin-modified screen-printed electrodes (SPCE) through grafting with p-aminobezoic acid (p-ABA) using EDC/Sulfo-NHS chemistry. The MWCNTs/GQDs hybrid nanomaterial was used as a nanocarrier of multiple detector antibodies and horseradish peroxidase (HRP) molecules, improving the sensitivity assay due to the intrinsic peroxidase-like activity of GQDs. The amperometric detection of the analyte, using the system H_2_O_2_/hydroquinone (HQ), allowed it to achieve a linear calibration range from 2.7 to 100 ng mL^−1^ of IL-13sRα2 and with a LOD value of 0.8 ng mL^−1^. The performance of this immunosensor was successfully applied to the determination of the target receptor in raw cellular lysates from different colorectal cancer cells and extracts of paraffin-embedded tissues from patients affected with colorectal cancer at different stages. In another work, the same group proposed a dual electrochemical immunoassay for the simultaneous detection of IL-13Rα2 and cadherin-17 (CDH-17) an important cancer biomarker involved in several tumor processes with different metastatic potential and also a sensitive marker for gastric adenocarcinoma [129]. In this work, the MWCNT/GQDs-functionalized screen-printed dual carbon electrode (SPdCE) was assembled to form a sandwich sensor [106]. Following the same detection strategy previously described, the authors reported for this dual amperometric immunosensor the selective detection of the biomarkers IL-13sRα2 and CDH-17, with respective LOD values of 1.4 ng/mL and 0.03 ng/mL, respectively, as evaluated for lysates from breast and colorectal cancer cells. 

The human chorionic gonadotropin (HCG) represents a diagnostic marker for the detection and monitoring of pregnancy, but it is also an extremely sensitive and specific marker for a variety of cancers including choriocarcinoma and extra-uterine malignancies [130]. Recently, Roushani et al. reported a label-free electrochemical immunosensor for the selective detection of HCG in human serum based on GQDs-N-S/Au modified SPCE [107]. In this study, nitrogen- and thiol-doped GQDs, synthesized from citric acid, were functionalized with amine and thiol groups after reaction with 3-aminopropyl-triethoxysilane and 3-mercaptopropyl-triethoxysilane. The nanomaterial was casted on the surface of a modified SPCE, conjugated with Au nanoparticles to afford the AuNPs/GQDs-N-S system and then functionalized with the HCG antibody. Electrochemical analyses performed on this immunosensor showed the high sensitivity of the sensor towards the investigated biomarker with a linear communication in the range of 0.1 to 125 pg mL^−1^ and a LOD of 12.5 fg mL^−1^. The same procedure was successfully employed also for the detection of HCG in human serum samples.

Immunosensors that combine nucleic acid-based systems, allow to overcome some limitations related with the classical immunosensors, namely the type of substances that can be detected and sometimes, the difficulty to achieve sufficient sensitivity without additional amplification steps. Moreover, the production of monoclonal antibodies used in the specific antibody–antigen interactions is a costly and time-consuming procedure and unfortunately, these macromolecules can loss their specific activity after denaturation, thus requiring mild reaction conditions during the experimentations [131]. To overcome these limitations, immunosensors which uses DNA or RNA probes able to bind directly to selected targets as bioreceptors or to act as signal amplifiers have been developed [132]. New sensing methodologies use as biorecognition elements DNA or RNA aptamers, short synthetic single chain DNA or RNA oligonucleotides, which fold into three-dimensional structures. These molecules are able to react specifically and with high affinity with different small molecules and proteins [133]. 

**Figure 6:**
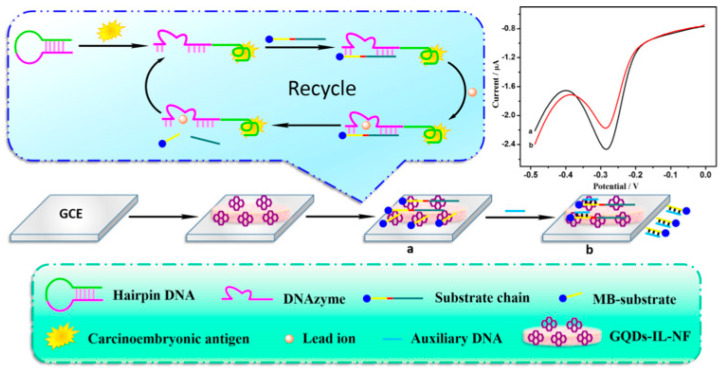
GQDs−IL−NF nanomatrix and DNAzyme−assisted recycling of target-aptamer complex for the sensitive electrochemical detection of CEA. Reprinted from Biosensors and Bioelectronics, ref [108], copyright (2018), with permission from Elsevier. (**a**) conjugation of GQDs with DNAzyme chain, (**b**) addition of MB substrate.

Aptamers that can recognize the biomarker CEA have been isolated and investigated for in vivo imaging and diagnosis of cancer cells [134]. The aptamer strategy towards CEA analysis was successfully investigated by Zhao et al. by developing a combination of GO and aptamer labeled CdSe/ZnS quantum dots, by capillary electrophoresis [135]. The authors reported a linear relationship in the fluorescence intensity with concentrations of CEA in the range from 0.257 to12.9 ng/mL, and a LOD of 5pg/mL. The high sensitivity and specificity of the nanosystems was also proved in serum of patients. An electrochemical aptasensor for the high-sensitivity determination of CEA based on GQDs/ionic liquid-nafion (GQDs-IL-NF) composite film as the substrate for the DNA immobilization and Pb^2+^-dependent DNAzyme assisted target recycling signal amplification, was developed by Wang et al. [108]. In this work, GQDs with sizes in the range of 1.5–5 nm, synthesized from pyrolysis of citric acid, were mixed with nafion and with the ionic liquid 1-butyl-2,3-dimethylimidazolium tetrafluoroborate and then drop-coated on the surface of GCE; the subsequent treatment with aptamer solution allowed them to obtain the CEA electrochemical aptasensor. The mechanism of action of this sensor is based on a hairpin DNA constituted by the CEA-specific aptamer sequence and by a DNAzyme chain (Figure 6). In the presence of CEA, the hairpin DNA recognized the antigen, thus forming the CEA/aptamer complex; the subsequent interaction with the sequence of DNAzyme linked with methylene blue (MB) allowed the introduction of a nick site in DNAzyme and in the presence of Pb^2+^ ions, the cleavage of the substrate chain into two fragments occurred. The CEA-aptamer complex was then released and the DNAzyme-assisted signal amplification reaction was performed, yielding a large number of MB substrates, thus producing the electrochemical signal. The authors reported a response current change proportional to the concentrations of CEA, with a linear range from 0.5 fg mL^−1^ to 0.5 ng mL^−1^, and a LOD of 0.34 fg mL^−1^. The aptasensor was also successfully applied in determining CEA in serum samples.

A sandwich–type electrochemical aptasensor for the sensitive detection of CEA was also developed by Zare et al. [109]. In this study, GQDs with sizes of ~5.7 nm obtained from pyrolysis of citric acid were electrodeposited on the surface of a GCE firstly modified with N–doped graphene and with gold nanoparticles.

The CEA-binding aptamer was then covalently linked to the surface of the electrode by amide formation between the carboxyl groups of GQDs and the amino groups of the aptamer. Subsequently, the amino modified CEA aptamer II (ApII) linked to DNAzyme through linkage with glutaraldehyde (GA) was placed on the electrode surface to form a sandwich structure. Subsequently, different concentrations of CEA solutions were incubated onto the surface of the biosensor and the hemin–G4 acted as peroxidase-mimicking DNAzyme, catalyzing the electroreduction of hydrogen peroxide. The quantitative determination of CEA was obtained by DPV showing a linear response in the range of 10.0 fg mL^−1^ to 200.0 ng mL^−1^ and a low LOD of 3.2 fg mL^−1^.

Exosomes are extracellular vesicles surrounded by a lipid bilayer membrane with a diameter range of 30–150 nm, and are secreted by almost all cells. These nano-vesicles are now considered as important mediators of intercellular communication, contributing to a wide range of biological processes, including cancer [136]. Exosomes are able to transfer bioactive macromolecules such as proteins, DNA, RNA, lipids and metabolites from the cells of origin to other cells. It was also demonstrated that these vesicles are more often released by cancer cells than healthy ones, thus leading to physiological changes and promoting tumor growth. Due to these interesting properties, exosomes have been recently investigated for the diagnosis and monitoring of cancer, as well as for the development of engineered vehicles for cancer treatment [137] Goreham et al. demonstrated the potential in using exosomes as nano-sized cancer biomarkers, using as detecting agent indium phosphide quantum dots conjugated with the CD63 antibody. This non-toxic conjugate demonstrated the ability to detect exosomes derived from a monocyte cell line (THP-1), expressing the protein CD63 [138]. Gold–carbon quantum dots (GCDs) conjugated with tumor-specific antibodies have been used for fluorescence imaging of exosomes, through immuno-reactions. The results of this study demonstrated the potential of this nanoprobe to investigate the intrinsic intracellular behavior of tumor derived exosomes [139]. Ramadan et al. developed graphene field-effect transistors decorated with carbon dots (CDs-GFET) and conjugated with CD-63 antibodies to target the CD-63 biomarkers present on the surface of the exosomes. The decoration with CDs have proved to allow a ultra-low LOD (100 particles/μL) [2]. The antibody of prostate-specific membrane antigen (PSMA) was recently used by Mahmoudifard et al. to label GQDs obtained from graphite in order to develop a fluorescent immunosensor able to detect prostate cancer (PCa) derived exosomes from blood serum [110]. In this study, the authors used a sandwich type process for the isolation and detection of exosomes, using a specialized nanofibrous membrane composed of polycaprolactone and gelatin which was conjugated with CD-63 antibodies and worked by capturing and holding exosomes during the washing processes, while the antibody conjugated GQDs identified the captured exosomes. The results obtained from photoluminescence studies showed a linear fluorescence quenching ability correlated to exosome concentration with a LOD of 7.5 mg ml^−1^. The system showed the ability to selectively recognize exosomes from mixtures containing different biomolecules and bio complexes, thus highlighting the potential of this approach for the immunosensing of cancer. 

### 4.3. Nucleic Acid Hybridization Sensors

In recent years, DNA hybridization sensors emerged as promising clinical diagnostic devices for the detection of cancer and infectious diseases. These biosensors mainly consist of single-stranded DNA (ssDNA) probes immobilized on a transducer surface, which after the recognition with the complementary DNA target form a DNA double helix. This hybridization process can be converted into a quantified signal by the transducer for the optical, electrochemical, piezoelectric or thermal detection [140]. A highly selective and sensitive fluorescence sensing approach for DNA detection based on GQDs and GO oxide was reported by Feng et al. [111]. In this study, the authors combined the strong fluorescence of GQDs synthesized by acidic oxidation of graphite, the base pairing specificity of the ssDNA conjugated with the GQDs and the fluorescence resonance energy transfer (FRET) between the GQDs and GO to achieve quantitative analysis of the DNA target. The authors reported for this system a broad linear range of 6.7–46.0 nM and a LOD of 75.0 pM DNA. Joshi et al. developed an electrochemical biosensor based on a simple screen-printed DNA sensor for the detection of the *p16* tumor-suppressor gene in prostate cancer [112]. In this work, GQDs synthesized from GO, were conjugated with the ssDNA sequence, and the subsequent hybridization with the ssDNA target was monitored by DPV and by electrochemical impedance spectroscopy (EIS). The system showed the ability to detect a single base mismatch in the presence of complementary and non-complementary oligonucleotide sequences, reporting a LOD of 0.10 pM. An electronic method for the quantification of the SEPT9 gene whose hypermethylation in the promoter region, is associated to the development of colorectal cancer (CRC), was recently reported by Jia et al. [141]. In this study, carbon dots synthesized by a one-step hydrothermal method starting from citric acid, have been drop-coated on the channel surface of liquid exfoliated graphene field effect transistor (LEG-FETs) to form the CD-modified LEG-FETs (CDs-LEG-FETs). Then, the target SEPT9 sequences were deposited on the system, allowing the recognition of the 5-methylcytosine (5 mC) positions by the respective antibody and the responses were transduced according to the immunologic recognition and FET’s sensing mechanism. The authors reported a detection sensitivity for the low-quantity of DNA samples as low as 2 ng. Chen et al. developed a signal-off photoelectrochemical biosensor for the evaluation of M.SssI methyltransferases (MTase) activity, an enzyme implied in DNA methylation and involved in cancer diseases [113]. In this study, zeolitic imidazolate framework-8 (ZIF-8) polyhedra were used as carriers of streptavidin labeled GQDs. Indium tin oxide (ITO) slice modified with TiO_2_, poly(diallyldimethylammonium chloride) and CdTe quantum dots (ITO/TiO_2_/CdTe QDs) was functionalized with ssDNA via S–Cd bond and used as photoelectrode. Then, after hybridization with biotinylated ssDNA, the streptavidin-labeled GQDs@ZIF-8 polyhedra were introduced to the photoelectrode surface through the specific reaction between biotin and streptavidin. The GQDs@ZIF-8 polyhedra were shown to inhibit the photocurrent signal of the ITO/TiO_2_/CdTe QDs electrode due to steric hindrance effect and also worked as peroxidase mimetics to catalyze the precipitation reaction of 4-chloro-1-naphthol, thus affording a depression in the photocurrent signal. This photocurrent decrease was quantitatively correlated with the enzyme activity with a linear response range, 0.005–150 U mL^−^^1^ and a LOD of 0.004 U mL^−^^1^.

MicroRNA (miRNA) is a small single-stranded RNA that plays an important role in different cellular processes including cancer progression [142]. An electrochemical biosensor based on a gold nanoparticles, GQDs and GO (AuNPs/GQDs/GO) for the detection of miRNA-21, miRNA-155, and miRNA-210 biomarkers in human serum was recently reported by Ounnunkad et al. [114]. In this work, the authors used three redox species (anthraquinone, methylene blue and polydopamine) to capture miRNA probes able to hybridize with the complementary targets, miRNA-21, miRNA-155, and miRNA-210, respectively. This multiplex label-free miRNA biosensor demonstrated excellent performance for the simultaneous miRNA sensing, also distinguishing the miRNA target from other interfering oligonucleotides. The authors reported a wide linear range of 0.001 to 1000 pM and the low LODs of 0.04, 0.33, and 0.28 fM for the detection of miRNA-21, miRNA-155, and miRNA-210, respectively. 

### 4.4. Circulating Tumor Cells Sensors

The early and selective diagnosis of circulating tumor cells (CTC) is an important challenge in oncotherapy since when cancer cells are released from the primary tumor into the bloodstream, they can be considered the main promoters of metastasis. Thus, their evaluation and quantification are important issues to understand the tumor biology and to improve the clinical treatment of cancer diseases [143]. The developed sensing methodologies are mainly based on the detection of specific receptors over-expressed on the surface of cancer cells to ensure their fast proliferation, and to cancer related byproducts such as H_2_O_2_ [144]. Yang et al. fabricated an electrochemical biosensor based on reduced graphene oxide quantum dots (rGO QDs)/ZnO hybrid nanofibers, for the detection of H_2_O_2_ released from cancer and normal cells, under the stimuli of anticancer drugs [145]. The authors demonstrated the ability of the system to quantify the amount of H_2_O_2_ released from a prostate cancer cell (PC-3) and healthy cells (BPH-1) under the stimuli of the anticancer drugs apigenin and with the antisense CK2a oligonucleotide. Wang et al. explored an electrocatalyst for the sensitive and specific detection of extracellular H_2_O_2_ released from living cancer cells, based on Pd nanoparticles decorated double shell structured N-doped GQDs@N-doped carbon (NC) hollow nanospheres (HNSs), for the sensitive and specific detection of extracellular H_2_O_2_ released from living cancer cells [115]. In this work, N-GQDs synthesized from the carbonization of citric acid, were wrapped on amino-functionalized SiO_2_ nanospheres through electrostatic and hydrogen bonds interactions and coated with polydopamine (PDA) via self-polymerization of dopamine hydrochloride on their surface. The subsequent decoration with Pd NPs followed by carbonization under inert atmosphere and etching of SiO_2_ cores with HF solution, allowed to obtain the sensing substrate NGQD@NC@Pd HNSs. This hybrid material demonstrated high electrocatalytic activity toward the electrochemical reduction of H_2_O_2_ with short response time (within 2 s) at the favorable potential of 0 V and with a low LOD (20 nM). The biosensor was successfully investigated for the real-time tracking of trace amounts of H_2_O_2_ secreted from different human living cells (MDA-MB-231 and HBL-100) and of the U87 cancer cell line. An ultrasensitive electrochemical biosensor based on multiple layer CdS QDs functionalized polystyrene microspheres (PS) as bioprobe and (GO)-polyaniline (PANI) as capture electrode, was developed by Wang et al. for the detection of K562 cell, a human immortalized myelogenous leukemia cell line [146]. The biosensor showed a low LOD of 3 cells mL^−1^ and a wide linear range (10–1.0 × 10^7^ cells mL^−1^), and was also successfully tested for mannosyl groups on HeLa cells and Hct116 cells, showing high specificity and sensitivity. 

**Figure 7:**
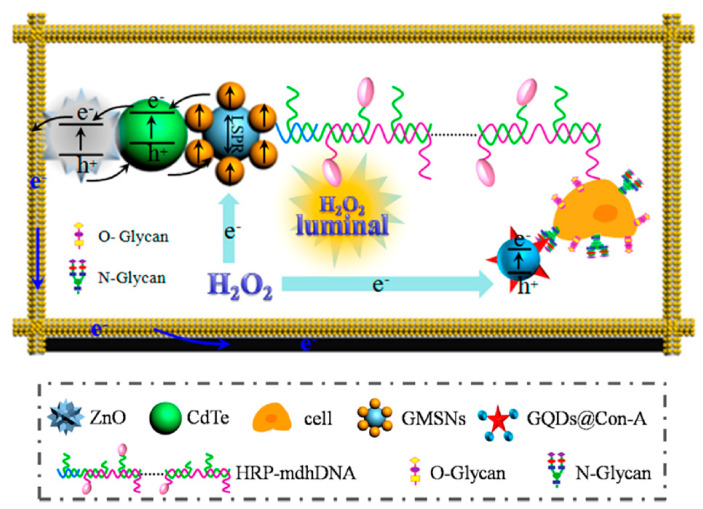
Analytical principle of photoelectrochemical biosensor based on ConA−GQDs. Reprinted with permission from ACS Applied Materials & Interfaces, ref. [116]. Copyright (2017) American Chemical Society.

Aberrant glycan epitopes are a classic hallmark of cancer and can be used to recognize and detect different kind of tumors [147]. Yu et al. reported an ultrasensitive photoelectrochemical biosensor for the N-glycan expression based on GQDs, synthesized by chemical oxidation from carbon black, nanogold-assembled mesoporous silica nanoparticles (GMSNs) and combined with multibranched hybridization chain reaction (mHCR) [116]. In this study, GQDs were conjugated with concanavalin A (ConA), a carbohydrate-binding protein able to recognize N-glycan expressed on the cancer cell surface. Porous ZnO spheres were covered on the paper working electrode with Au nanorod (Au-PWE) and with CdTe QDs and subsequently with GMSNs; then, the HRP labeled DNA with multiple branched arms (HRP-mdhDNA) was covalently linked with the ethanediamine treated GMSNs through the formation of amide bond with the carboxyl group of the enzyme. The analytical principle of this biosensor is depicted in Figure 7. The chemiluminescent (CL) emission of this luminol-HRP-H_2_O_2_ system used HPR as inner light source able to excite the photoactive materials. Under the light source, ZnO sensitized by CdTe QDs and with the aid of the localized surface plasmon resonance of GMSNs, gave photocurrent response. The interaction of ConA-GQDs with the cancer cells competitively absorbed the CL, producing a weakening in the photocurrent intensity. The authors reported that, when compared with control tests, the biosensor showed greater ability to evaluate N-glycan expression of the cancer cells surface with high sensitivity and with a LOD of 21 cells per mL.

A “turn-on” magnetic fluorescent biosensor based on N,S/GQDs Fe_3_O_4_, and MoS_2_ nanosheets for the rapid and sensitive detection of circulating tumor cells was reported by Cui et al. [117]. In this study, the photoluminescent GQDs with an average diameter of 5 nm were synthesized by electrolysis of graphite rods using a solution of sodium p-toluenesulfonate. The nanomaterials were conjugated via EDC/Sulfo-NHS chemistry with a complex constituted by the magnetic agent Fe_3_O_4_ and a functional aptamer able to target the epithelial cell adhesion molecule (EpCAM). The system was coupled with MoS_2_ nanosheets, which acted in this system as fluorescence quenchers. This “turn-on” magnetic fluorescent nanocomposite was investigated in different cultures of low- and high-EpCAM-expressing cancer cell lines (Hep G2, A549, HEK293), showing a specific ability to quickly identify CTCs (both low- and high-EpCAM-expressing cells), because of the presence of aptamers, with a linear range between 2 and 64 nM, and a LOD of 1.19 nM, thus highlighting its potential for the for early diagnosis and prognosis of cancer. 

## 5. Conclusions and Future Remarks

The best chance to reduce cancer deaths and to increase the chances of treatment success is the early detection of cancer, possibly in the site of origin, before it invades the surrounding tissues and distant organs. The currently used molecular-based assays for the routine diagnosis of cancer, generally suffer from low sensitivity, time-consuming processes and the impossibility to allow the on-site detection, in biological media. The advances of nanotechnology in biosensing field has demonstrated great promises in revolutionizing cancer diagnostics, by empowering miniaturized biosensors for the effective on-site early diagnosis of cancer. Among the differently investigated nano-sized substrates for the detection of cancer biomarkers, the zero dimensional GQDs, showed great promises due to their remarkable electronic and optical properties, their large surface area and the presence of different active sites for the chemical functionalization. Their covalent bonding with biomolecules able to recognize cancer biomarkers or to detect tumor cells, have shown to afford advanced sensing substrates for the fast and accurate point of care cancer diagnosis and the monitoring of treatment progression. 

In this review, we have discussed different strategies involving the synthesis and functionalization of GQDs with different biomolecules able to recognize and convert into a signal, specific cancer biomarkers such as antigens, enzymes, hormones, proteins, cancer related byproducts, biomolecules exposed on the surface of cancer cells and also changes in pH. The developed biosensors have shown to ensure the effective diagnosis and monitoring of cancer diseases as well as the possibility to evaluate the effectiveness of anticancer therapy, thus suggesting their great potential in clinics for the diagnosis and management of cancer. However, despite the outstanding performances of the developed biosensors, the future research in this field will of course take into account the need to move biosensors into point of care systems for the detection of multiple cancer biomarkers. The devices should be sensitive, rapid, easy to use, also ensuring the accuracy and reliability of the laboratory. 

The major challenge in using GQDs for the fabrication of sensing devices is the large-scale synthesis of high-quality and stable nanoparticles, with definite size, shapes, charge and agglomeration state, since these features have a great impact on the physicochemical properties of these nanomaterials and thus, on the GQDs based sensors performance. The outstanding results obtained in the use of these nanomaterials for immunosensing still require more studies in order to compare the obtained results with other existing techniques. The excellent chemical, physical and optical properties of GQDs will allow many application in biosensing field. However, new protocols for the detection of cancer biomarkers and cancer cells are needed for these highly sensitive immunosensors. We believe that the use of a multidisciplinary approach that combines knowledge in biology, physics, chemistry and engineering, will certainly guarantee the possibility, in the next future, to develop smart biosensors able to provide the easy, fast and accurate early diagnosis of cancer, thus increasing the patients’ survival rates.

## Figures and Tables

**Table 1:** Summary of typical approaches for the synthesis of GQDs as biosensing platforms.

Approach	Method	Source	Ref.
Top-down	Hydrothermal	graphite	[47]
		graphene	[48]
		graphene oxide	[49,50]
		corn powder	[51]
		cellulose	[52]
		rice husk	[53]
	Liquid exfoliation	graphite	[54,55,56,57]
	Electrochemical	graphite	[58,59,60]
		graphene oxide	[61]
		wood charcoal	[62]
Bottom-up	Hydrothermal	pyrene	[63]
		pyrene and polyethyleneimine	[64]
		1,3,6-trinitropyrene and borax	[65]
		citric acid	[66,67]
		glucose	[68,69]
		starch	[70]
		urea and glucose	[71]
	Template methods	1,3,5-triamino-2,4,6-trinitrobenzene	[72]
		hexa-peri-hexabenzocoronene	[73]
		carbon disuphide	[74]
	Pyrolysis	L-glutamic acid	[75]
		citric acid	[76]
		trisodium citrate	[77]
	Irradiation methods	salicylic acid and pyridine-2,6-dicarboxylic acid	[78]
		glucose	[79]
		1,3,6-trinitropyrene	[80]

**Table 2:** Summary of some recent GQDs-based biosensors for cancer detection.

Sensing Material	Biological Material	Analyte	Detection Technique	Performance	Ref.
GQDs	L929, HT-1080, MIA, PaCa-2, HeLa, MG-63 cells	pH	optical	−49.96 ± 3.5 mV/pH	[94]
S-N-doped GQDs	mice bearing PANC-1, A549 HepG2, U87MG, HeLa cells	pH	optical	switch point at pH 6.8	[95]
GQDs-DNPTYR	MCF-7 cells	H_2_S	optical	LOD: 2 nM.	[96]
RBD-GQDs	Pancreatic CSCs, HeLa cells	Fe^3+^	optical	LOD: 0.02 μM	[97]
GQDs-cAb	-	CA-125	chemiluminescence	LOD: 0.05 U mL^−1^	[98]
Ag–DPA–GQDs	plasma	CA-125	electrochemical	LOD: 0.001 U mL^−1^	[99]
PtPd/N-GQDs@Au	serum	CEA	electrochemical	LOD: 2 fg/mL	[100]
GQDs@Au	serum	CEA	electrochemiluminescence	LOD: 3.78 fg/mL	[101]
N,S-GQDs@Au-PANI	serum	CEA	electrochemical	LOD: 0.01 ng/mL.	[102]
amine-N-GQDs@Au	serum	NSE	optical	LOD: 0.09 pg mL^−1^	[39]
CysA/Au NSs/GQDs	plasma, MCF-7 cells	CA 15-3	electrochemical	LOD: 0.11 U/mL	[103]
P-Cys-GQDs-GNPs	plasma, L929, HCT PC-3, MCF-7 cells	p-53	electrochemical	LOD: 0.065 fM	[104]
MWCNTs/GQDs	lysates from colorectal cancer	IL-13Rα2	electrochemical	LOD: 0.8 ng mL^−1^	[105]
MWCNTs/GQDs	lysates from breast and colorectal cancer	IL-13Rα2 CDH-17	amperometric	LOD: 1.4 ng/mL (IL-13sRα2); 0.03 ng/mL (CDH-17)	[106]
GQDs N-S/Au	serum	HCG	electrochemical	LOD: 12.5 fg mL^−1^	[107]
GQDs-IL-NF	serum	CEA	electrochemical	LOD: 0.34 fg mL^−^^1^	[108]
GQD/AuNP/NG/	serum	CEA	electrochemical	LOD: 3.2 fg mL^−1^	[109]
GQDs	serum	PCa- exosomes	optical	LOD: 7.5 mg ml^−^^1^	[110]
GO/GQDs	-	DNA	optical	LOD: 75.0 pM	[111]
GQDs	-	*p16* tumor-suppressor gene	electrochemical	LOD: 0.10 pM	[112]
GQDs@ZIF-8	-	M.SssI MTase	photoelectrochemical	LOD: 0.004 U mL^−^^1^	[113]
AuNPs/GQDs/GO	serum	miRNA	electrochemical	LOD: 0.04 fM (miRNA-21), 0.33 fM (miRNA-155), 0.28 fM (miRNA-210)	[114]
NGQD@NC@Pd HNSs	MDA-MB-231, HBL-100, U87 cells	H_2_O_2_	electrochemical	LOD: 20 nM	[115]
ConA-GQDs	MCF-7 cells	N-glycan	electrochemiluminescence	LOD: 21 cells/ mL	[116]
Apt@Fe_3_O_4_@GQDs/MoS_2_	Hep G2, A549, HEK293 cells	EpCAM	optical	LOD: 1.19 nM,	[117]
